# Prevalence and trends of major congenital anomalies in Brazil: A study from 2011 to 2020

**DOI:** 10.1371/journal.pone.0323654

**Published:** 2025-06-06

**Authors:** Mariana Pereira Inácio Silvestri Melkan, Ornella Scardua Ferreira, Livia Carolina Leonardo Bassan, Maria de Lourdes Brizot, Rossana Pulcineli Vieira Francisco, Agatha Sacramento Rodrigues, Mariana Azevedo Carvalho

**Affiliations:** 1 Department of Obstetrics, Faculdade de Medicina FMUSP, Universidade de Sao Paulo, Sao Paulo, Brazil; 2 Department of Statistics, Universidade Federal do Espírito Santo UFES, Espírito Santo, Brazil; Federal University of Sergipe, BRAZIL

## Abstract

**Background:**

Congenital anomalies contribute significantly to morbidity and mortality among newborns and infants. In Brazil, the estimated prevalence of malformations in newborns is < 1%, which is comparatively lower than that recorded in other regions worldwide. This study aimed to analyze the prevalence of congenital anomalies in Brazil over a 10-year period and to identify potential associations of this prevalence with socioeconomic, gestational, and regional factors by performing an analysis using data sourced from the Live Birth Information System (*Sistema de Informações sobre os Nascidos Vivos* – SINASC) covering the period from 2011 to 2020.

**Methods:**

From a total population of 29,025,461 live births, we included a cohort of 240,405 newborns with congenital anomalies. For the purpose of this study, we categorized newborns with congenital anomalies into two groups: one group with newborns with a single major malformation and another group with newborns with multiple major malformations (minor malformations not considered).

**Results:**

The prevalence of congenital anomalies was 8.0 per 1,000 live births, with variations across different years and regions within the country. The Southeast region of Brazil, with the highest human development index, displayed the highest prevalence of congenital anomalies. The most frequent congenital anomalies were limb deformities (29.7%), neural tube defects (14.7%), and heart defects (11.6%).

**Conclusion:**

The prevalence of major congenital anomalies in Brazil during the study period varied with the geographic region and was lower than that in developed nations, likely due to lower prenatal detection rates and underreporting.

## Introduction

Congenital anomalies are defined by the World Health Organization as “structural or functional anomalies that occur during intrauterine life” [1]. These anomalies can be diagnosed at birth or throughout life and are attributed to various factors such as monogenic disorders, chromosomal disorders, exposure to environmental factors (teratogens), or deficiency in micronutrients [1,2].

Globally, an estimated 3%–6% of children are born with congenital anomalies, and this number may fluctuate in correlation with the Gross Domestic Product per capita [1]. Nonetheless, irrespective of economic status, these anomalies significantly contribute to child morbidity and mortality worldwide [1]. Annually, at least 3.3 million children aged <5 years die due to congenital anomalies, and 3.2 million survive with sequelae [3]. Furthermore, these disorders have psychological and socioeconomic impacts on patients, their family members, and healthcare structures [1].

While most studies on the prevalence and trends of congenital anomalies have been conducted in developed countries [4–6], the prevalence of congenital anomalies in underdeveloped and developing countries must also be evaluated to determine whether poor health conditions affect the occurrence of congenital anomalies [7–9].

In Brazil, despite the requirement for healthcare professionals to complete the Certificate of Live Birth (*Declaração de Nascido Vivo* – DNV), less than 1% of live births (LBs) are annually documented to be born with a congenital anomaly, indicating underreporting compared with global estimates [10]. In Brazil, although DNV records offer the potential to analyze the prevalence and risk factors for congenital anomalies, no studies on trends regarding malformations in Brazil have been reported in the literature, which are crucial for informing preventive, diagnostic, and therapeutic interventions. Therefore, this study aimed to analyze the prevalence of congenital anomalies in Brazil from 2011 to 2020 using publicly available data of the Live Birth Information System (*Sistema de Informações sobre os Nascidos Vivos* – SINASC), provided by the Ministry of Health (*Ministério da Saúde* – MS).

## Methods

This study is part of the Brazilian Obstetric Observatory (*Observatório Obstétrico Brasileiro* – OOBr) project [11], which serves as a platform for evidence-based monitoring, analysis, and reporting of maternal and child health data in Brazil. As our analysis used publicly available database, this study did not require approval by a research ethics committee.

The microdata used in this study were extracted from the SINASC databases. SINASC, the official system of MS, is designed to record structural congenital anomalies detected either at birth (in the delivery room) or diagnosed prenatally in all newborns across the country. SINASC has been collecting data on births in Brazil recorded in DNVs since 1990, including data on structural congenital anomalies, using the International Statistical Classification of Diseases and Related Health Problems 10^th^ Revision, Clinical Modification (ICD-10-CM) code range for Chapter XVII Congenital malformations, deformations, and chromosomal anomalies Q00 – Q99 as reference [12]. Some of the microdata was previously analyzed by the Data Science Platform Applied to Health (*Plataforma de Ciência de Dados aplicada à Saúde* – PCDaS) based on the three-phase [Extract, Transform, and Load (ETL)] computing process. We retrieved the remaining microdata using the R {microdatasus} package [13].

For this study, we analyzed SINASC data spanning from 2011 to 2020. The selection process involved identifying all LBs recorded with an International Classification of Diseases (ICD) code for malformation (D180, Q00–Q99), as monitored by SINASC [14]. Among them, we excluded cases involving a single ICD code for a minor malformation from the analysis, as they have minimal medical and functional consequences. Minor malformations were defined according to the European Surveillance of Congenital Anomalies (EUROCAT) guidelines [15]. Major malformations were defined as malformations involving significant medical, social, and esthetic consequences for the individual, generally requiring medical intervention [16]. In addition, we excluded prenatally diagnosed chromosomal anomalies from the analysis, following the EUROCAT guidelines [15], and also because some aneuploidies are not associated with malformations, such as approximately 25% of Trissomy 21 cases [17]. Consequently, the study focused on examining two groups: newborns with a single malformation and newborns with multiple malformations.

Within the cohort exhibiting multiple congenital anomalies, we assessed the number and specific types of malformations present in each child. Cases of multiple malformations belonging to the same congenital anomaly class or whose second malformation stemmed from the first were included in the group with a single malformation. Subsequently, we also analyzed congenital anomalies based on anatomical location (central nervous system (CNS), heart, face, digestive system, abdominal wall, urinary system, genitals, and limbs), using the EUROCAT guide as reference [15].

Descriptive statistical analysis was conducted to present maternal characteristics (ethnicity/color, level of education, and marital status), gestational factors (number of prenatal visits and type of pregnancy), delivery details (gestational age and type of delivery), and newborn attributes (sex) as absolute and relative frequencies. Prevalence was calculated as the ratio of the number of LBs with at least one of the congenital anomalies of the specific groups to the total number of LBs documented in SINASC during the study period in the geographical area of the pregnant women, multiplied by a factor of 1,000 LBs. Statistical analysis was conducted using the free software environment for statistical computing and data visualization R [18], version 4.3.0 (R Foundation for Statistical Computing. Vienna, Austria).

Log-linear regression analysis was used to estimate the Annual Percent Change (APC) and the Average Annual Percent Change (AAPC) to assess overall prevalence trends of specific groups of congenital anomalies and their respective statistical significance during the period from 2011 to 2020. For this purpose, the year of birth was considered as the regressor variable, with a significance level of 5%.

## Results

**Fig 1:**
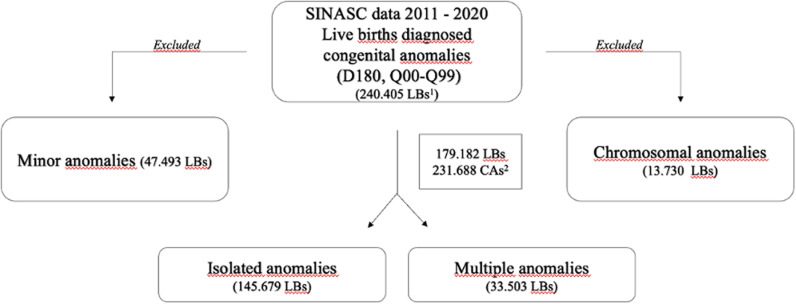
Study flow diagram. LBs: live births; CAs: congenital anomalies.

During the study period, out of 29,025,461 LBs in Brazil, we identified 240,405 newborns with congenital anomalies, accounting for 0.83% of LBs [19]. Among them, 13,730 (5.7%) had an ICD code for chromosomal abnormality, while 47,493 (19.8%) were found to have “minor” malformations, resulting in a final sample size of 179,182 newborns. Most (81.3%, n = 145,679) newborns showed a single malformation, while the remaining (33,503 newborns) showed multiple malformations (Fig 1).

**Fig 2:**
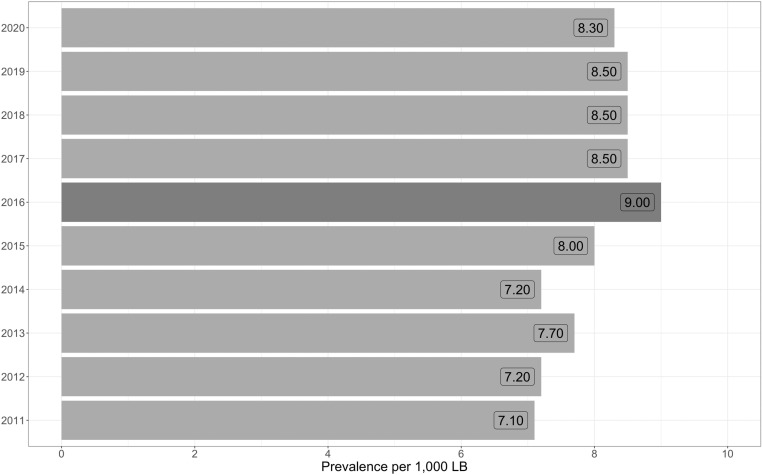
Prevalence of congenital anomalies by year, per 1,000 live births. LBs: live births.

**Fig 3:**
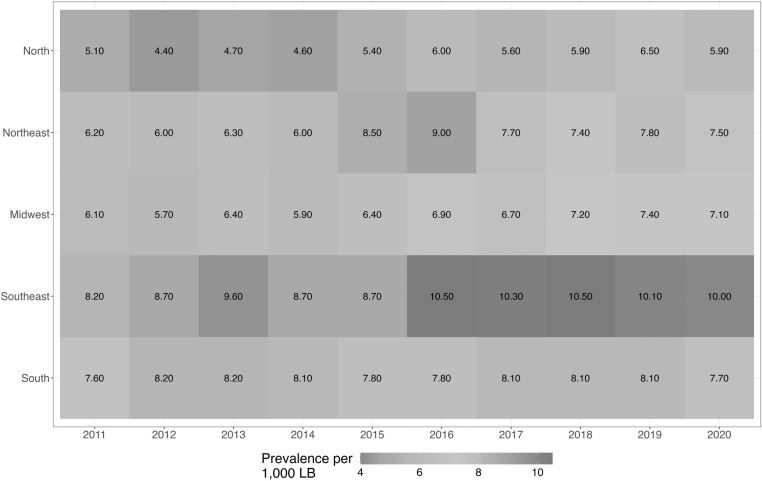
Prevalence of congenital anomalies by region, per 1,000 live births. LBs: live births.

Fig 2 illustrates the prevalence of congenital anomalies relative to the total number of LBs per year throughout the study period. The highest prevalence was recorded in 2016, reaching a rate of 9.0 per 1,000 LBs. The Southeast region, which has the highest human development index (HDI) in the country [20], showed the highest prevalence of congenital anomalies (Fig 3). However, the Northeast region also showed high prevalence in 2015 and 2016, in contrast to that in other years. In the statewide analysis, São Paulo, which is the state with the highest HDI in Brazil [20], almost invariably had the highest prevalence of congenital anomalies during the study period (S1 Fig).

**Fig 4:**
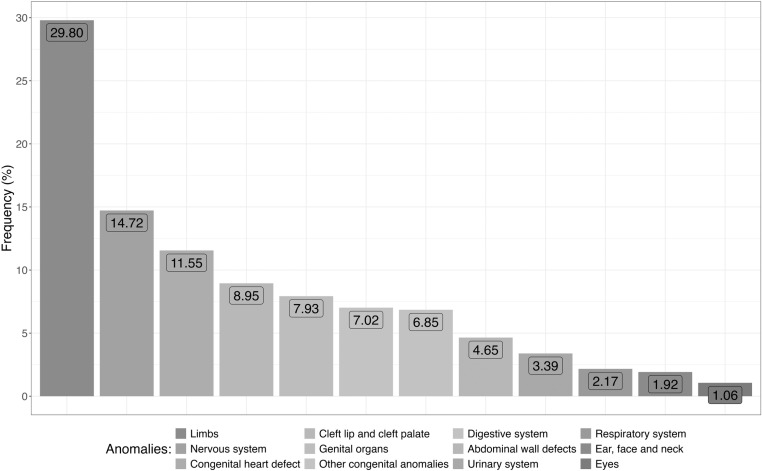
Frequency of congenital malformations in Brazil by group of anomalies.

The analysis based on group and type of malformation showed that the most frequent congenital anomalies were limb deformities (29.7%), neural tube defects (14.7%), and heart defects (11.6%) (Fig 4), and that polydactyly (9.9%) was the most frequent congenital malformation, followed by supernumerary fingers (5.5%), and hypospadias (3.3%) (S2 Fig).

Table 1 outlines the overall and group-specific prevalence of congenital anomalies according to maternal, gestational, and newborn characteristics. The prevalence of congenital anomalies was higher among male newborns and in twin pregnancies than among female newborns and in singleton pregnancies. We also observed a similar number of cases classified as unspecified sex in the data records (n = 3362), which is comparable to the number of cases categorized as indeterminate sex according to the ICD (n = 3434). Furthermore, the general prevalence of congenital anomalies was also higher in mother’s of black ethnicity, as it was the prevalence of central nervous system malformations. In addition, the prevalence of neural tube defects decreased with an increase in education level.

**Table 1:** Prevalence of major congenital anomalies in Brazil categorized according to specific groups and maternal, pregnancy, and newborn characteristics, per 1.000 live births.

Variable	Category	Number of live births diagnosed with CA^1^	Number of live births	General prevalence of CA	Groups of congenital anomalies (CA)							
					Central nervous system	CHD^2^	Cleft lip and cleft palate	Digestive system	Abdominal wall defects	Urinary system	Genital organs	Limbs
Delivery type	C-section^3^	114781	16222292	7.08	1.37	1.06	0.69	0.60	0.47	0.30	0.66	2.19
	Vaginal	64008	12765175	5.01	0.66	0.55	0.56	0.37	0.17	0.18	0.47	2.07
Mother’s ethnicity	Asian	772	110182	7.01	1.01	2.23	0.69	0.65	0.36	0.31	0.48	1.91
	White	62661	9574676	6.54	1.00	1.27	0.73	0.59	0.35	0.33	0.62	1.93
	Native	1195	227707	5.25	1.09	0.34	0.76	0.54	0.23	0.06	0.35	1.56
	Mixed	87865	15072997	5.83	1.09	0.58	0.59	0.44	0.33	0.19	0.55	2.18
	Black	12091	1535530	7.87	1.18	1.05	0.63	0.52	0.38	0.30	0.71	3.31
NB gender	Female	75303	12706457	5.93	1.23	0.90	0.60	0.48	0.37	0.16	0.09	2.12
	Male	100517	13335324	7.54	1.11	0.96	0.80	0.60	0.37	0.36	0.91	2.60
Mother’s marital status	Married	57260	9495480	6.03	0.96	1.07	0.65	0.52	0.21	0.29	0.59	1.97
	Separated/divorced	2343	336409	6.96	1.03	1.25	0.71	0.60	0.27	0.33	0.63	2.38
	Single	79442	12547678	6.33	1.12	0.75	0.63	0.48	0.42	0.22	0.57	2.25
	Consensual marriage	38178	6258987	6.10	1.09	0.64	0.63	0.51	0.38	0.22	0.56	2.19
	Widowed	363	52254	6.95	0.86	0.67	0.71	0.54	0.52	0.36	0.57	2.74
Mother’s scolarity	None	1011	171695	5.89	1.20	0.27	0.86	0.57	0.20	0.09	0.53	2.18
	1-3 years	5187	852126	6.09	1.19	0.39	0.69	0.51	0.25	0.17	0.56	2.33
	4-7 years	33126	5333350	6.21	1.16	0.49	0.68	0.51	0.41	0.19	0.56	2.28
	8-11 years	105330	16833266	6.26	1.09	0.80	0.63	0.48	0.36	0.25	0.58	2.23
	12 years or more	32795	5375997	6.10	0.86	1.43	0.63	0.55	0.23	0.32	0.59	1.76
Number of fetuses	One	173113	28372943	6.10	1.05	0.82	0.63	0.49	0.34	0.24	0.56	2.13
	Two	5518	591092	9.34	1.52	1.57	0.75	0.81	0.52	0.31	1.14	2.55
	Three or more	172	14714	11.69	1.43	4.55	0.95	1.16	0.41	0.68	1.09	2.92
Gestational age at birth	< 22 weeks	265	14884	17.80	5.85	1.21	0.60	0.60	1.14	0.54	4.97	2.35
	22-27 weeks	3111	141564	21.98	5.83	3.87	1.86	2.00	1.46	1.14	2.01	4.83
	28-31 weeks	6923	294177	23.53	5.46	3.45	2.05	2.92	1.90	1.96	2.68	5.23
	32-36 weeks	35131	2758124	12.74	2.62	1.65	1.15	1.39	1.47	0.85	1.30	3.31
	37-41 weeks	126931	24230480	5.24	0.81	0.73	0.56	0.37	0.20	0.16	0.47	1.98
	>= 42 weeks	3812	838421	4.55	0.81	0.35	0.55	0.34	0.13	0.09	0.39	1.94
Number of prenatal care appointments	None	4187	640326	6.54	1.63	0.51	0.60	0.58	0.40	0.20	0.60	2.08
	1-3	14197	1859221	7.64	1.67	0.71	0.72	0.63	0.57	0.28	0.70	2.56
	4-6	45391	6953201	6.53	1.25	0.64	0.66	0.55	0.46	0.24	0.62	2.27
	7 or more	113674	19373233	5.87	0.90	0.93	0.62	0.46	0.27	0.24	0.54	2.05

^1^CA: congenital anomalies;

^2^CHD: congenital heart disease;

^3^C-section: cesarean section delivery.

The overall prevalence trends of specific groups of congenital anomalies are described in Table 2. All groups exhibited an increasing trend over the years of this study, with a statistically significant increase of prevalence in the following groups: congenital heart diseases, abdominal wall defects, urinary system, genital organs and limbs. In this specific case, the AAPC is equal to the APC, because the trend was analyzed within a single time frame (from 2011 to 2020).

**Table 2:** Average Annual Percent Change and prevalence trends according to specific groups of congenital anomalies during the period from 2011 to 2020.

Groups of congenital anomalies	AAPC^1^	95% CI	P – value
Central nervous system	1.52	(-4.38; 7.79)	0.577
Congenital heart disease	6.92	(3.72; 10.22)	0.001
Cleft lip and cleft palate	0.63	(-0.42; 1.68)	0.204
Digestive system	0.45	(-0.64; 1.54)	0.372
Abdominal wall defects	1.38	(0.27; 2.50)	0.021
Urinary system	2.94	(1.32; 4.59)	0.003
Genital organs	1.33	(0.47; 2.19)	0.007
Limbs	1.15	(0.19; 2.12)	0.024

^1^AAPC: Average annual percent change.

## Discussion

The main findings of this evaluation of 10-year major congenital malformation records in Brazil can be summarized as follows: the prevalence of congenital anomalies in Brazil is comparatively lower than that in developed nations but is higher in regions with a higher HDI (Southeast and South); the most frequent congenital anomalies are limb deformities, neural tube defects, and heart defects; and the prevalence of congenital anomalies was higher in the Northeast region during the Zika virus outbreak.

This study provides a contemporary overview of congenital anomalies in Brazil, focusing on data collected during 2011–2020. This timeframe was selected, as it aligned with the implementation of the current notification system in 2011, ensuring consistency and reliability in the data collection process. Brazil has a lower prevalence of congenital anomalies than developed countries (8.0 per 1,000 LBs in Brazil vs. 19.1 per 1,000 LBs in Europe) [21], primarily because of underreporting and failure to include diagnosed cases until hospital discharge. The prevalence of congenital anomalies increased during this 10-year period, particularly of congenital heart diseases, abdominal wall defects, urinary system malformations, genital organs anomalies and limb malformations. This can be explained by improvements in the notification system and diagnosis rates (resulting from the adoption of advanced technology and enhanced training of qualified healthcare professionals).

Regarding prevalence based on region (Fig 3), an increase was observed in the number of malformation cases in the Northeast region in 2015 and 2016. This increase most likely resulted from the outbreak of microcephaly caused by the Zika virus in the region during this period [22]. Additionally, when analyzing differences between Brazilian regions, we noted a higher number of notifications in the Southeast and South regions, possibly due to the higher socioeconomic status of the inhabitants of these regions and, therefore, due to higher rates of prenatal diagnosis, as opposed to diagnosis occurring solely at birth [23,24]. Van der Linde et al., in 2011, also observed that regions with higher HDIs showed a higher prevalence of malformations [25]. Another contributing factor to increased malformation rates in the Southeast of Brazil could be the migration of pregnant women to this region for childbirth, given the presence of hospitals experienced in monitoring newborns with complex congenital anomalies, such as heart defects [26–29].

Some studies have also associated high rates of congenital anomalies with increased exposure to pollutants [30]. Accordingly, the least populated regions and those with few large urban centers, such as the North, Midwest, and Northeast regions, have the lowest rates of congenital anomalies, potentially due to their lower levels of pollution. Nevertheless, even inland regions (i.e., outside large urban centers) are exposed to pollution resulting from sugarcane plantations [31] and slash-and-burn agriculture [32], which is prevalent in Brazil.

Regarding maternal and gestational characteristics during the study period, 56% of all newborns in Brazil were delivered via cesarean section, whereas 44% were delivered vaginally [33]. The proportion of newborns with congenital anomalies who were delivered via cesarean section is even higher, accounting for 64.2% of cases. In Brazil, where cesarean birth rates remain high, even in situations where vaginal birth is preferable [34], higher cesarean birth rates among fetuses with congenital anomalies may be due to high maternal or fetal risk during vaginal birth. In addition, not all healthcare centers and professionals are willing to provide adequate care for pregnancies with fetal malformations. A study conducted in the United States by McCormick et al., in 2019, showed that cesarean birth rates were higher in pregnancies in which the fetus had congenital malformations [35].

We also observed differences in the prevalence of congenital anomalies between different ethnicities with a higher prevalence of congenital anomalies among black women (7.87 per 1,000 LBs), with emphasis on CNS malformations (1.18 per 1,000 LBs). There are conflicting results in the literature regarding the prevalence of congenital anomalies among different racial groups. Schraw et al., in 2024, reported that the distributions of maternal race/ethnicity was similar among cases of congenital anomalies [36]. In contrast, other studies have demonstrated significant differences among malformations groups; for example, a study conducted by Ray et al., in 2004, fround that the prevalence of neural tube defects was higher among women of First Nations origin [37].

Analysis of the prevalence of neural tube defects, or CNS malformations, based on the maternal education level exhibited an inverse association: the lower the maternal education level, the higher the prevalence of neural tube defects. Pregnant women with lower education levels often reside in poor socioeconomic conditions and face an increased risk of unwanted and unplanned pregnancies [38]. The higher prevalence of neural tube defects in this group of pregnant women may be associated with low rates of folic acid supplementation and nutritional deficiency [39], which are risk factors for the development of neural tube defects during pregnancy [39,40]. Furthermore, Brazil being the epicenter of the Zika virus outbreak in 2015 and 2016, as mentioned earlier, significantly contributed to increased rates of microcephaly. A low maternal education level is one of the risk factors for this viral infection [41].

A higher prevalence of congenital heart diseases was positively associated with the number of clinical appointments, of 0.5 per 1,000 LBs in the group of no clinical appointments and 0.93 per 1,000 LBs in the group of 7 or more appointments. This association is most likely explained by the increased access to fetal morphological ultrasonography and echocardiography in the group with better prenatal care, which leads to higher rates of prenatal diagnosis. Furthermore, many congenital heart diseases may not present specific symptoms in newborns while they are still in the delivery room [42], when the notification of congenital anomalies is done. This underscores the critical importance of prenatal diagnosis in increasing the survival prospects of these newborns [43]. Brazil has a lower prevalence of congenital heart diseases than Europe (0.93 vs. 5.7 per 1,000 LBs) [44] and even in São Paulo, which has the highest HDI in the country, this value remains at 2.42 per 1,000 LBs, thus showing the need for improving prenatal care throughout the country.

This study has some limitations. First, there is only access to live birth data and not to fetal death data. Accumulation of information on fetal death is crucial, as some types of congenital anomalies increase the risk of intrauterine death [45]. Furthermore, abortion law is stricter in Brazil than in developed countries, allowing termination of pregnancy only in cases where the fetus is diagnosed with anencephaly [46]. Consequently, legal termination of pregnancy has limited impact on the overall prevalence of congenital anomalies. A second limitation is that SINASC monitors only within Chapter XVII (D180, Q00 – Q99) codes, and this code range excludes codes for congenital infections like toxoplasmosis and cytomegalovirus, despite their high prevalence [47,48].

The primary strength of this study is that it is the first to analyze the prevalence of congenital malformations in Brazil over an extended period. Another strength is the inclusion of data from all LB across the Brazilian territory, whereas many published studies encompass only a portion of the country’s population. Lastly, there are few published studies on the prevalence of congenital malformations in middle- and low-income countries, and this study contributed to assessing the differences in the prevalence of congenital malformations in countries with marked socioeconomic differences.

Some practical implications of our study include the significance of performing fetal morphological ultrasonography, which detects 70%–80% of malformations, for all pregnant women [49,50]. However, this examination is not mandated by the Brazilian MS in low-risk pregnancies [51] and consequently, the majority of pregnant women treated in the public health system do not undergo this examination. Conversely, if this examination is made mandatory for all pregnant women, the detection rates of congenital anomalies would increase, enabling healthcare professionals to more rigorously evaluate the prevalence and trends of these changes, particularly in cases of anomalies that are not visible on physical examination.

Another factor that would improve the assessment of this prevalence is adequate completion of DNV, which could be achieved by the use of digital platforms. This method would mitigate human error, such as inaccurate description of ICD codes, and would enable healthcare professionals to include diagnoses post-birth. Currently, the data captured in DNV is limited to information gathered during the prenatal period and in the delivery room.

## Conclusion

This study suggests that the lower prevalence of congenital anomalies in Brazil than in developed countries is likely attributable to lower prenatal detection rates and underreporting. Therefore, congenital anomalies must be constantly monitored because these data contribute to policymaking aimed at improving prenatal detection rates and enhancing care for newborns affected by congenital diseases.

## Supporting information

S1 Fig Prevalence of congenital anomalies by Brazilian state, per 1,000 live births. LBs: live births.
(TIF)

S2 Fig Frequency of congenital malformations in Brazil by anomaly ICD-10. CID-10: ICD-10 (International Classification of Diseases 10th Revision).
(TIF)
