# Supplementary figures and images for: Prevalence and trends of major congenital anomalies in Brazil: A study from 2011 to 2020
Source: PLoS One. 2025 Jun 6;20(6):e0323654. doi: 10.1371/journal.pone.0323654 (PMC12143536; doi:10.1371/journal.pone.0323654)

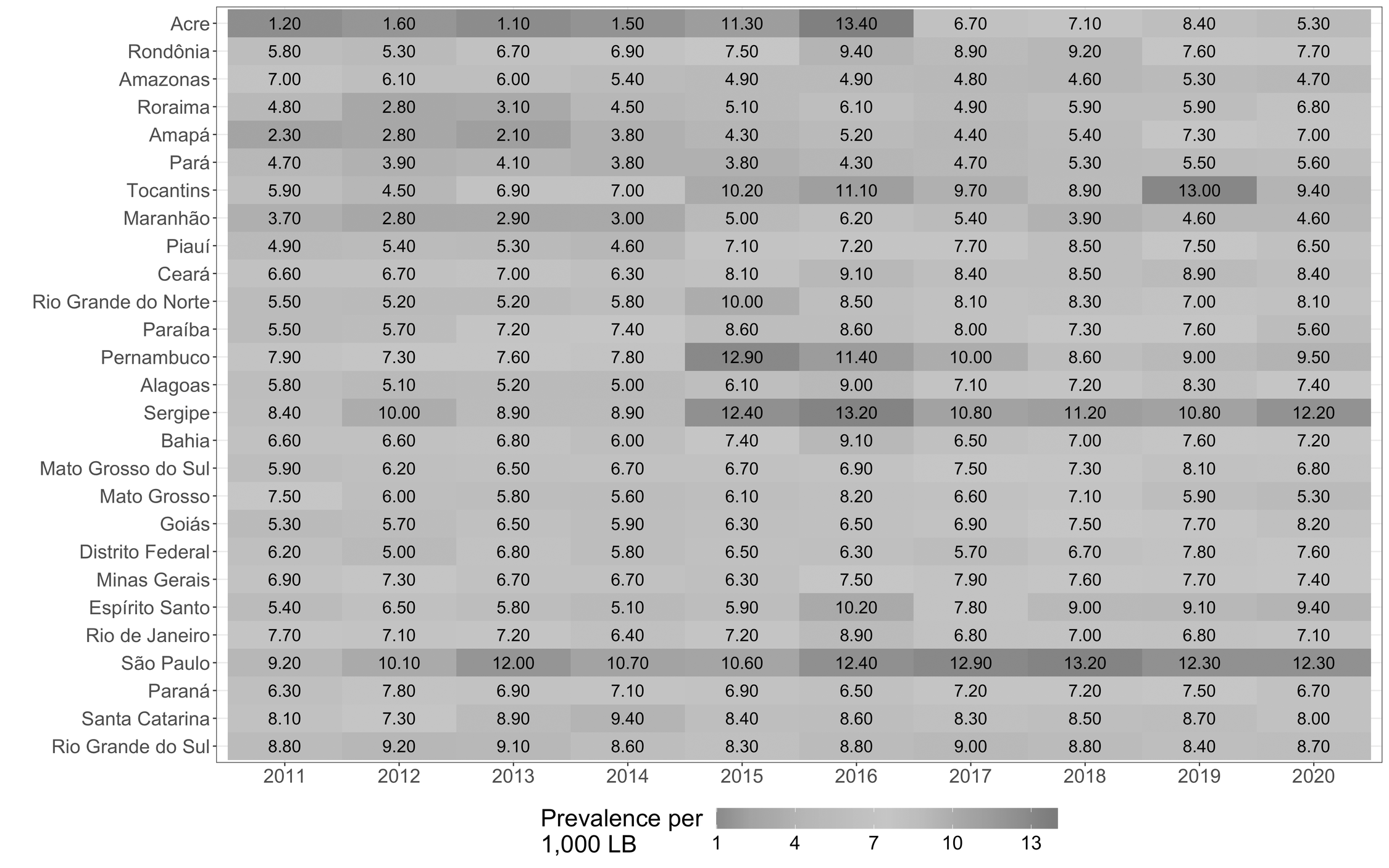

Supplement: S1 Fig — LBs: live births. (TIF) [file pone.0323654.s001.tif]

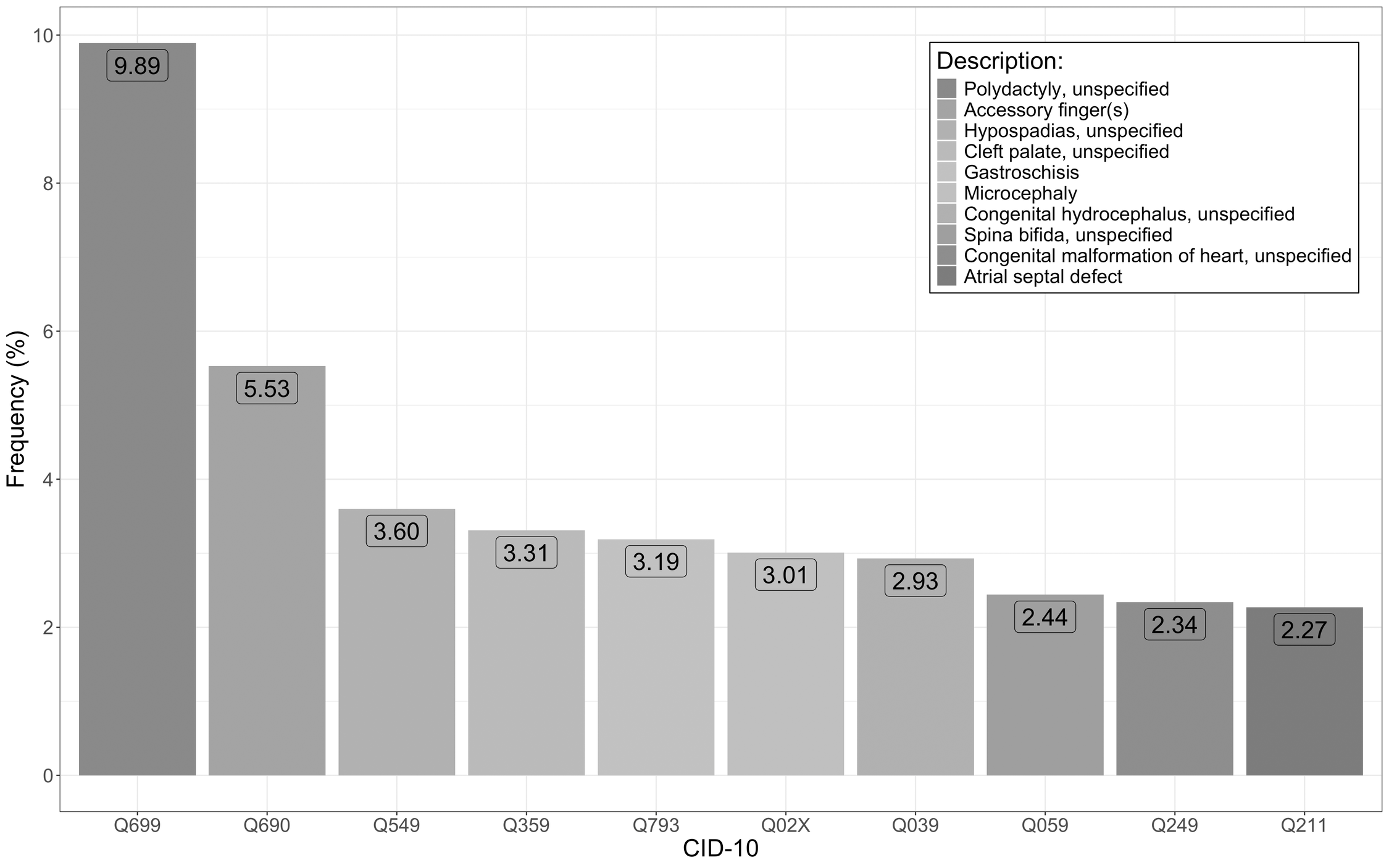

Supplement: S2 Fig — CID-10: ICD-10 (International Classification of Diseases 10th Revision). (TIF) [file pone.0323654.s002.tif]
